# Effect of Thickener Type on Change the Tribological and Rheological Characteristics of Vegetable Lubricants

**DOI:** 10.3390/ma17163959

**Published:** 2024-08-09

**Authors:** Rafal Kozdrach

**Affiliations:** Lukasiewicz Research Network—Institute for Sustainable Technologies, 26-600 Radom, Poland; rafal.kozdrach@itee.lukasiewicz.gov.pl; Tel.: +48-365-42-41-264-314

**Keywords:** lubricating grease, anti-wear characteristics, anti-scuffing characteristics, dispersed phase, limiting load of wear, limiting pressure of seizure, montmorillonite, modified silica aerosil, lithium stearate, aluminium stearate, calcium stearate, rheological properties, viscoelastic properties, yield point, viscosity curve, flow curve

## Abstract

This paper presents the results of a study on the effect of the dispersed phase on the lubricating and rheological properties of selected lubricant compositions. A vegetable oil base (rapeseed oil) was used to prepare vegetable lubricants, which were then thickened with lithium stearate, calcium stearate, aluminum stearate, amorphous silica, and montmorillonite. Based on the results of the tribological tests of selected lubricating compositions, it was found that calcium stearate and montmorillonite have the most beneficial effect on the anti-wear properties of the tested lubricating greases, while silica thickeners (amorphous silica and montmorillonite) provide the effective anti-wear protection in compared to the lubricants produced on lithium and aluminum stearate. The lowest structural viscosity was found for grease thickened with montmorillonite. Much higher values of this parameter were observed for composition, where aluminum stearate was the dispersed phase, while the highest value of structural viscosity was observed for composition, where aerosol–amorphous silica was the thickener. The composition thickened with amorphous silica had the highest yield point value, while the composition in which montmorillonite was the dispersed phase had the lowest value. Dynamic viscosity decreases with temperature, which is characteristic of lubricants. No significant differences in dynamic viscosity were found for the lubricating compositions tested at temperatures above 50 [°C]. The most favorable rheological properties were observed for composition, which was produced using calcium stearate, as it allows the lowest dynamic viscosity at −20 [°C]. Lubricants produced with lithium stearate or aluminum stearate were characterized by higher viscosity at low temperatures. For grease, in which the lithium stearate was used as a thickener, the value of the elasticity index determines the weak viscoelastic properties of tested grease and a greater tendency to change structure under the influence of applied forces. For vegetable grease thickened with aluminum stearate, more than 15 times lower values of the MSD function were observed, and the calculated elasticity index value proves the stronger viscoelastic properties of the aluminum stearate grease in relation to grease thickened with the lithium stearate. The elasticity index value for grease thickened with amorphous silica was lower than for greases thickened with lithium and aluminum stearate, indicating its stronger viscoelastic properties in relation to these two greases. For grease composition prepared on the vegetable oil base and thickened with montmorillonite. The value of the elasticity index was lower than most of the tested grease compositions, without the composition, in which the calcium stearate was used as a thickener. Such results testify to moderately strong viscoelastic properties, which leads to the conclusion that the produced lubricant was a stable substance on changes in chemical structure under the influence of variable conditions prevailing during work in tribological joints.

## 1. Introduction

Environmental protection has become increasingly important in recent years. EU regulations and legislation place great emphasis on limiting the use of environmentally harmful petroleum lubricants [1,2,3]. The search is therefore on for components with high biodegradability and non-toxicity. These requirements are met by components of vegetable origin, which are increasingly used in the production of ecological greases [4,5,6,7,8]. The non-toxicity and biodegradability of greases are particularly desirable when these products are used in food processing equipment [9,10].

Applications in the above-mentioned industry require the production of greases with suitably selected components, in particular non-toxic oil bases, which ensure the ecological character of the final product [11,12,13,14].

Currently, there is a growing interest in the possibility of using vegetable oils for the production of lubricants, mainly for environmental reasons. There is a growing trend to replace poorly biodegradable petroleum products that pollute the environment with non-toxic vegetable products. Vegetable oils have very good viscosity temperature and lubricating properties which determine their suitability as an oil base for lubricants. The main disadvantages of these products are low resistance to hydrolysis and, in particular, low thermal and oxidation resistance [15,16,17,18,19,20,21,22].

Lubricating greases are substances consisting of a dispersing phase, a dispersed phase, and additives. The dispersed phase usually consists of simple and complex lithium, sodium, aluminum, and calcium soaps, as well as bentonite, polyurethanes, silica and paraffin, and waxes and polymers. The content of the dispersed phase in a lubricant is usually between a few and several tens of percent [23,24,25].

The amount and nature of the dispersed phase determine the operating characteristics of the lubricant composition. In the manufacture of lubricating grease, a substance is selected that will impart appropriate tribological, rheological, corrosive, or mechanical properties to the resulting product [26]. In recent years, inorganic substances have been increasingly used as the dispersed phase of greases, which are characterized by excellent load-carrying capacity, high melting point, improved lubricating efficiency, reduced wear, and increased chemical and thermal resistance of the resulting lubricant [27,28,29,30]. The use of complex soaps as thickeners for lubricating greases increases the lubricant dropping point and improves mechanical and structural resistance and tribological properties.

One of the components of lubricating greases is a thickener. It is a substance that provides the spatial structure and appropriate rheological and tribological properties of the resulting grease. The amount of thickener in grease ranges from 5 [%] to 20 [%], depending on the type of thickener and the consistency of the grease [1,2,3]. A number of performance characteristics of greases such as texture, mechanical stability, temperature resistance, rheological properties, or resistance to water and acids depend on the type of used thickener [4].

A common classification of lubricating greases is based on the type of used thickener. On this basis, a distinction is made between soap greases (e.g., based on lithium stearate), mixed greases (e.g., based on lithium calcium soaps), inorganic greases (e.g., based on silica or bentonite), and polymer greases (e.g., based on polyurethanes or terephthalates) [31,32,33,34,35]. Today, complex and organic thickeners are becoming increasingly important in the manufacture of lubricating greases.

On the other hand, thickeners of organic origin, such as salts of metals from groups I and II of the periodic table and high molecular weight fatty acids are strong oxidizers and have high thermal stability, and greases thickened with these substances have long lifetimes [36,37,38]. Inorganic thickeners, which may include colloidal silica, are also important. They improve the chemical stability and the lubricating properties of the produced lubricating compositions, thanks to which there is an increased interest in this type of thickener on the world markets [39,40,41,42].

The research carried out on vegetable lubricants will allow scientific knowledge on the tribological and rheological behavior of vegetable lubricants to be expanded. The use of thickeners with a different chemical structure may influence the changes in the rheological and tribological properties of vegetable lubricants in different ways.

The aim of this study was to investigate the effect of different thickeners used in the same base oil on the modification of the basic tribological parameters of lubricating compositions applicable in the food industry.

## 2. Materials and Methods

A series of model lubricant compositions were developed using non-toxic ingredients as dispersion and dispersed phases. Rapeseed oil (Komagra, Warsaw, Poland)was used in the dispersing phase. The rapeseed oil used to prepare grease compositions was defined by the specific physicochemical parameters as follows 0.853 [g/cm^3^] density; 36.41 [cSt] kinematic viscosity at 40 [°C]; 2.51 [meq O_2_/kg] peroxide number; 98.11 g [I_2_/100 g] of iodine index; 234.83 [mg KOH/g] of saponification index; and 0.88 [mg KOH/g] of acid index. The base oil used to prepare the vegetable lubricants was refined [43,44].

Lithium stearate (Merck, Darmstadt, Germany) [31], calcium stearate (Merck, Darmstadt, Germany) [16], aluminum stearate (Merck, Darmstadt, Germany) [40], Aerosil^®^-type modified silica (Evonik Corporation, Parsippany, NJ, USA) [33], and montmorillonite (Heiltropfen, London, UK) [45] were used as dispersed phases. The selected components were used to produce vegetable lubricants of the second consistency class, which can be used in the food industry. The consistency class of the produced lubricant compositions was tested in accordance with the requirements of the PN-ISO 2137:2021 [46] standard using a laser penetrometer manufactured by LRN-IST-ITeE [31,33]. Table 1 shows the composition of the tested lubricants. The following designations were used for the compositions prepared on the basis of rapeseed oil and thickened with lithium stearate (grease A), aluminum stearate (grease B), calcium stearate (grease C), modified silica Aerosil^®^ (grease D), and montmorillonite (grease E).

Different amounts of dispersed phase (7–12 [% m/m]) were introduced into the lubricant compositions used in the experiment. The different levels of thickener in the lubricants tested were due to the fact that it was assumed that the lubricating greases produced would be in the second consistency class (penetration value must be 265–295 [mm/10]). At an early stage of the test, the amount of thickener to be added to the base oil was investigated in order to obtain lubricating compositions falling within the second consistency class. Trials were carried out with compositions containing between 5 and 15 [%] in the dispersed phase. The lubricating compositions prepared in this way were then subjected to tribological tests.

A T-02 four-ball tester [47,48,49] was used to determine the tribological properties of the tested lubricating compositions. A Nikon MM-40 (Nikon Metrology Inc., Tokyo, Japon) optical microscope was used to determine the wear scar diameters of the test ball surfaces. The results obtained were used to determine G_oz/40_ and p_oz_ values, i.e., to evaluate the anti-wear and anti-scuffing properties of the lubricating greases subjected to tribological tests [33,45,46,47,48,49].

The tribological properties of the tested lubricants were determined by measuring the limiting load of wear (G_oz/40_), welding load (P_z_), scuffing load (P_t_), limiting a load of scuffing (P_oz_), and limiting pressure of seizure (p_oz_) [33,50]. The test specimens were 12.7 [mm] diameter balls made of ŁH 15 bearing steel, surface roughness Ra = 0.32 [µm], and hardness 60–65 [HRC]. The limiting load of wear (G_oz/40_) was measured when the friction node was loaded with a force of 392.4 [N] for the entire test duration of −3600 [s] and at a ball speed of 500 [rpm] according to the test conditions specified in WTWT-94/MPS-025 [51].

The limiting load of wear is a parameter that measures the lubricant’s anti-wear properties. The measurement of this property was carried out by evaluating its value according to the equation [33,50]:

G_oz_ = 0.52 ∗ P_n_/d_oz_^2^ (1)

where:

P_n_—load of the tribosystem equal to 392.4 [N],
d_oz_—the diameter of the scars on the steel balls used for the test.

Measurement of welding load was performed according to PN-76/C-04147 [33,49,50]. This determination consisted of 10-s runs of an assembly of four balls in the presence of lubricating greases under increasing load until the balls were welded together. On the other hand, the measurement of the lubricating properties under scuffing conditions (i.e., under continuously increasing load during the test run) was carried out according to the methodology developed by LRN-IST. The test was performed with a linearly increasing load from 0 to 7200 [N] for 18 [s] at a spindle speed of 500 [rpm] and a load build-up rate of 409 [N/s]. When there is a sudden increase in frictional torque, the load level of the node is referred to as the scuffing load P_t_ [33,50].

Measurements were performed until a limiting frictional torque of 10 Nm or a maximum device load of 7200 [N] was reached. This point was defined as the limiting load of scuffing P_oz_. The arithmetic mean of at least three determinations not differing by more than 10 [%] was taken as the final result. The Q-Dixon test was used to statistically process the results at the 95 [%] confidence level.

The limiting pressure of seizure is a measure of the anti-scuffing properties of lubricants under scuffing conditions. The determination of this parameter consisted of calculating its value according to the formula: p_oz_ = 0.52 ∗ P_oz_/d_oz_^2^, where P_oz_ is the limiting load of scuffing and d_oz_ is the diameter of the defect formed on the steel balls used in the test [33,49,50].

The uncertainty in the determination of the quantities tested (limit load of wear G_oz/40_, welding load P_z_, scuffing load P_t_, limiting load of scuffing P_oz,_ and limiting pressure of seizure p_oz_) was estimated on the basis of the accuracy class of the used measuring apparatus. The correlation of the individual results of the quantities tested, i.e., G_oz/40_, P_z_, P_t_, P_oz,_ and p_oz_, and the dataset was verified using a Q-Dixon test at a 95 [%] confidence level.

An Anton Paar MCR-101 air-bearing rotational rheometer was used to determine the rheological properties (flow and viscosity curves) of the tested vegetable lubricants. The Rheoplus software (21 CFR Part 10) was used to control the rheometer and analyze the measurement data.

The tests were carried out using a cone-plate measuring system. A zero gap adjustment was then required for the selected measuring system, which was carried out at the test temperature. This is performed automatically by the instrument. Once the zero gaps had been set, a sample (lubricant) was placed in the center of the plate in such a quantity that it was evenly distributed over the surface of the cone when the cone head was lowered into the measuring position. This is normally 5 [g]. The test temperature was then determined. It was decided to perform the tests at ambient temperature, i.e., 20 [°C]. The shear rate range over which the tests were carried out (0.01 ÷ 100 [s^−1^]) and the measurement intervals at which the data were recorded (number of measurement points, total measurement time, and frequency of data collection) were then determined. A logarithmic curve relevant to the determination of the yield point was used to measure the flow curves. Tests were carried out on 5 grease samples prepared using different thickeners.

Viscosity curves (the dependence of viscosity from temperature or shear rate) were determined for the same greases on the same apparatus at a constant shear rate of 100 [s^−1^] over a wide temperature range (−20 ÷ 180 [°C]) and at a constant temperature of 20 [°C] in the shear rate range 0 ÷ 1000 [s^−1^] [33,45,46,47,48,49,52].

To describe the flow curves of the tested vegetable lubricants, the Casson rheological model was used, which takes into account the yield point and allows the highest correlation coefficient for lubricants to be obtained.

The Casson model describes the flow curves of non-linear plastic-viscous fluids (including lubricants) as follows [53,54]:

τ12=τ012+η∞ γ

where:

*τ*—shear stress [Pa],
*τ*_0_—yield point [Pa],
*η*_∞_—structural viscosity of grease [Pa*s],
*γ*—shear rate [s^−1^].

Linear regression was used to determine the value of η_∞_ and τ_0_^1/2^ and the correlation coefficient was then determined. Uncertainty in the determination of the test quantity was verified using the Q-Dixon test at the 95% confidence level [53,54,55,56,57,58,59,60].

The tests of the rheological properties of lubricants were carried out using the optical rheometer of DWS RheoLab (Swiss Company LS Instruments AG, Freiburg, Switzerland). The device uses Diffusing Wave Spectroscopy (DWS) to determine the rheological characteristics of the media, such as suspensions, emulsions, oils, greases, and foams. The working principle of the DWS rheometer is based on the assumption that light transport can be treated as a diffusion process in optically turbid samples. The apparatus provides microrheological measurements of materials in conditions of static intermolecular displacements in a wide range of frequencies and viscoelasticity [45,61].

The optical rheometer provides tests in two different geometries, i.e., transmission and backscattering [EU Patent 1720000] [62]. In the transmission mode, scattered light is detected after passing through the sample, and intensity fluctuations are measured. However, in the backscattering mode, light is collected and scattered back towards the incident beam, and its fluctuations are measured [45,63,64,65].

The correlation function mean square displacement (MSD), G′ modulus, and G″ modulus, under static conditions, were carried out. The rheological measurements were carried out at temperatures of 293 [K]. On the base of the analysis of appointed rheological parameters, the change in viscoelastic characteristics of lubricant samples with different thickener types was carried out. In order to carry out correct tests, the experiments affecting the calibration of the device, the selection of the measurement mode, and the thickness of the cuvette, were carried out. Before starting the measurements, the device was calibrated using a standard, which is an emulsion of polystyrene with a particle size of 222 [nm] in water. Next, the refractive indices for the oil base of particular lubricants were appointed, the time of the measurement was appointed, and the size of the spectrometer cuvette in which the samples were placed was prepared by adding a marker (titanium dioxide with a particle size of 360 [nm]) [45,61,66,67,68,69]. The selection of the cuvette for the tested media is very important because the correct measurements require fulfilling the condition that the ratio of the cuvette thickness (L) to the mean free path of particle (l*) must be greater than 7 and less than 30, otherwise the measurement is not possible [70,71,72]. The measurements were carried out in the transmission mode and the backscattering mode in order to determine the best optical path and to obtain correct results [73,74,75]. Then, the samples were homogenized and placed in a measuring cuvette. The rheological tests will be carried out in a measuring cuvette with an optical path equal to 1 [mm]. To statistically evaluate the results, the Q-Dixon test was used with a confidence level of 95 [%].

## 3. Results and Discussion

Below are the results of tribological tests (anti-scuffing and anti-wear properties) of lubricating compositions prepared with rapeseed oil and thickened with lithium stearate, aluminum stearate, calcium stearate, modified Aerosil^®^ silica, and montmorillonite.

**Figure 1:**
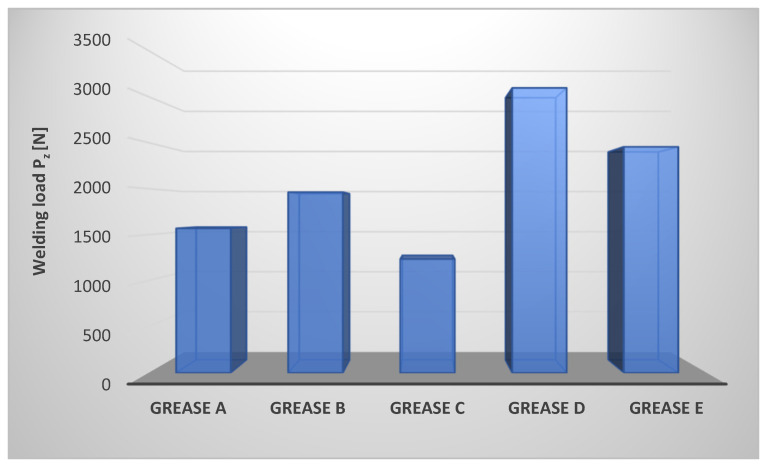
Welding load of a tribosystem lubricated with compositions based on rapeseed oil with different thickeners.

The welding load P_z_ was determined for the selected lubricating compositions. The results are shown in Figure 1.

The anti-scuffing properties of the lubricating greases tested under abruptly increasing friction loads depend on the nature of the dispersed phase, which has a significant effect on the durability of the lubricating film (Figure 1). The most favorable anti-scuffing properties were found for a composition in which the amorphous silica Aerosil was used as a thickener (grease D) and for a composition in which the dispersed phase was layered aluminosilicate–montmorillonite (grease E). On the other hand, the lowest anti-scuffing properties were obtained with a composition in which calcium stearate was used as the dispersed phase (grease C). Lubricating compositions based on the rapeseed oil thickened with lithium stearate (grease A) and aluminum stearate (grease B) are characterized by intermediate values of the determined parameter. The value of the welding load P_z_ for composition D (thickened with amorphous silica) is 150 [%] higher than the value of this parameter for composition C, in which calcium stearate was used as the dispersed phase). Thus, the analysis shows that the most effective anti-scuffing protection with a stepwise increase in the load on the friction node is represented by the lubricating composition in which Aerosil–amorphous silica was used as a thickener, while the least effective anti-scuffing action was found for the composition in which calcium stearate was used as the dispersed phase.

**Figure 2:**
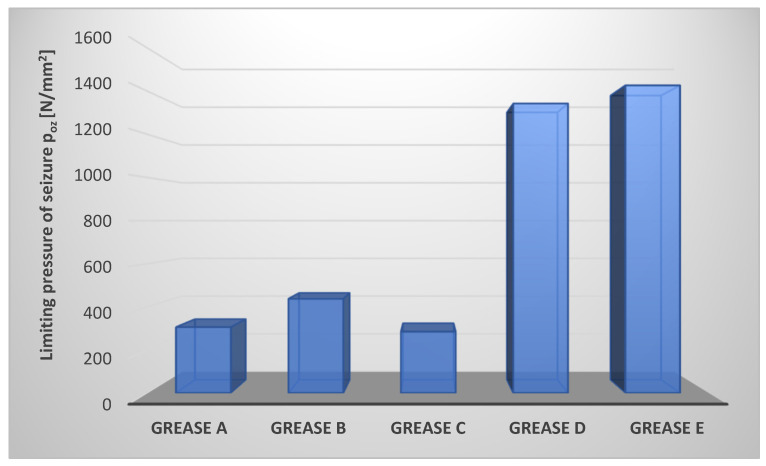
Limiting pressure of seizure of tribosystem lubricated with compositions based on rapeseed oil with different thickeners.

A measure of the anti-scuffing properties of the tested lubricants under seizure conditions is the limiting pressure of seizure p_oz_. The test results obtained for this parameter are shown in Figure 2.

The determined values of the limiting pressure of seizure showed different levels of anti-scuffing properties of the tested grease compositions. Depending on the used thickener, the value of the parameter determining the level of anti-scuffing properties for the tested lubricating greases changed significantly. The composition E, where the montmorillonite-containing layered aluminosilicates were used as the dispersed phase, was characterized by the highest value of the p_oz_ parameter. Composition D, where Aerosil–amorphous silica was the dispersed phase, is characterized by a high value of the p_oz_ parameter similar to composition E. The lowest value of this parameter was observed for grease C, where calcium stearate was the dispersed phase. The compositions A, B, and C are characterized by comparable values of the limiting pressure of seizure p_oz_, which reflects the level of their anti-scuffing properties. The value of the limiting pressure of seizure is p_oz_ for composition E (thickened with montmorillonite) is more than four times and for composition D (thickened with amorphous silica) almost four times higher than the value of this parameter for composition C, in which the calcium stearate was used as the dispersed phase.

Lubricating compositions prepared from rapeseed oil and thickened with lithium stearate, aluminum stearate, and calcium stearate did not show such favorable anti-scuffing properties as compositions in which the modified amorphous silica–Aerosil and montmorillonite—were used in the dispersed phase (Figure 2). The values determined for the limiting pressure of seizure showed that the use of amorphous silica and layered silicate as thickeners in vegetable lubricants has the most favorable effect on increasing the resistance of the surface layer to seizure than the use of soap thickeners in the tested lubricating compositions in the experiment. The p_oz_ parameter provides information about the pressure prevailing in the friction zone at the moment of seizure. From the results obtained, it can be concluded that the use of montmorillonite and amorphous silica in the composition of vegetable lubricants influences the formation of highly seizure-resistant surface layers. In contrast, the use of soap thickeners in the formulation of lubricants does not affect the formation of anti-scuffing surface layers. The higher p_oz_ parameter compared to compositions thickened with lithium stearate or calcium stearate for a lubricating composition in which aluminum stearate was used as the dispersed phase indicates that the nature of the film formed favors the reduction of wear.

Thus, the analysis shows that the most effective anti-scuffing protection of the friction node is represented by the lubricating composition, in which the montmorillonite, a representative of layered aluminosilicates, was used as a thickener, while the least effective anti-scuffing effect was found for the composition in which the calcium stearate was used as a dispersed phase.

**Figure 3:**
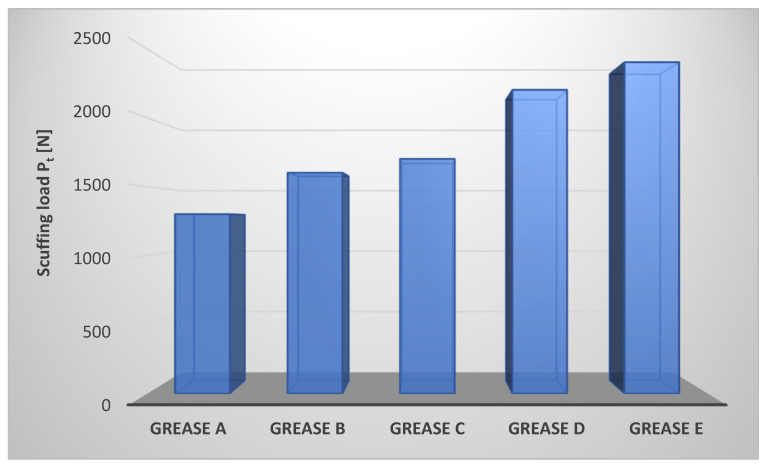
Scuffing load of tribosystem lubricated with compositions based on rapeseed oil with different thickeners.

For all prepared lubricating compositions, the anti-scuffing properties were determined under linearly increasing load conditions, characterized by the scuffing load P_t_. The obtained results are shown in Figure 3.

The scuffing load determines the level of anti-scuffing properties of the tested lubricants under linearly increasing load conditions. The properties characterized by the P_t_ parameter determine the load-carrying capacity of the lubricating film. The value of the anti-scuffing load varied according to the type of dispersed phase used in the manufacture of the lubricating greases. The most favorable anti-scuffing properties under conditions of linearly increasing load are characterized by a lubricating composition based on rapeseed oil thickened with montmorillonite (composition E). Composition D, in which amorphous silica–Aerosil was the dispersed phase, is characterized by a comparable value of the P_t_ parameter to composition E. The lowest value of this parameter was observed for lubricant A, in which the lithium stearate was used as a dispersed phase. Compositions B and C are characterized by the same value of the scuffing load P_t_, which determines the level of their anti-scuffing properties under a linearly increasing tribosystem load. The value of the scuffing load for composition E was 84.6% and for composition D was 69.2 [%] higher than the value of this parameter for composition A, in which the lithium stearate was used as a dispersed phase.

Thus, the type of dispersed phase used in the composition of vegetable lubricants influences the change of the anti-scuffing properties of the tested lubricating compositions. The lubricating compositions based on rapeseed oil with soap thickeners did not show such favorable anti-scuffing properties as compositions thickened with montmorillonite and modified amorphous silica–Aerosil (Figure 3). The greatest durability of the lubricating film is ensured by the use of amorphous silica and montmorillonite as the dispersed phase of the produced lubricating compositions, which makes it possible to obtain greases with the highest P_t_ parameter value. It can, therefore, be assumed that the anti-scuffing effectiveness depends on the structure of the boundary layer formed by the used thickeners. The individual particles of the dispersed phase in the lubricating film are more closely packed, which increases their interactions and thus increases the resistance of the lubricating film to higher loads.

**Figure 4:**
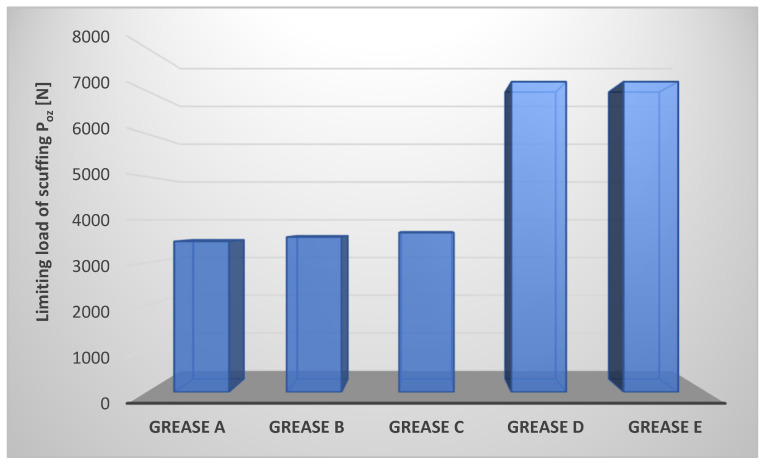
The limiting load of scuffing of tribosystem lubricated with compositions based on rapeseed oil with different thickeners.

The limiting load of scuffing of the tribosystem lubricated with the tested lubricating compositions was also determined. The results are shown in Figure 4.

The limiting load of scuffing allows the level of anti-scuffing properties of the tested greases to be determined. The most favorable anti-scuffing properties are characteristic of greases in which the modified amorphous silica and montmorillonite—a representative of layered silicates (compositions D and E)—were used as a dispersed phase. The lowest value of this parameter was observed for lubricant A, where the lithium stearate was used as a dispersed phase. The compositions A, B, and C are characterized by comparable values of the P_oz_ parameter-limiting load of scuffing, which determines the level of their anti-scuffing properties. The values of the limiting load of scuffing for compositions D and E were 105.7 [%] higher than the value of this parameter for composition A, in which the lithium stearate was used as a dispersed phase. Thus, the type of dispersed phase used in the composition of vegetable lubricants has a strong influence on the variation of the anti-scuffing properties of the studied lubricating compositions. The compositions prepared from rapeseed oil and thickened with lithium stearate, calcium stearate, or aluminum stearate did not show such favorable anti-scuffing properties as those in which the montmorillonite and modified amorphous silica were used as a dispersed phase (Figure 4).

The most effective effect of the lubricant after breaking the lubricating film is ensured by using amorphous silica and montmorillonite as thickeners of lubricating compositions since these thickeners make it possible to obtain greases characterized by the highest value of the P_oz_ parameter. For the compositions in which soap thickeners were used as dispersed phase (greases A, B, and C), the values of the P_oz_ parameter are in the 3500–3700 [N] range, which may indicate that the differences in the composition of the tested lubricating compositions play a significant role only under conditions of moderate forcing. In the case of compositions prepared with silicone thickeners (amorphous silica and montmorillonite), the limiting load of scuffing reaches a value of 7200 [N], which may indicate that the studied lubricating compositions effectively protect the friction node from seizure under high forcing conditions. During the seizure process, the increasing pressure in the friction zone means that there is no longer a lubricating film on the cooperating surfaces. A protective effect against seizure of the friction node can be provided by the used lubricating compositions, which can react with the friction pairing material. This has the effect of reducing the possibility of adhesion seizure.

**Figure 5:**
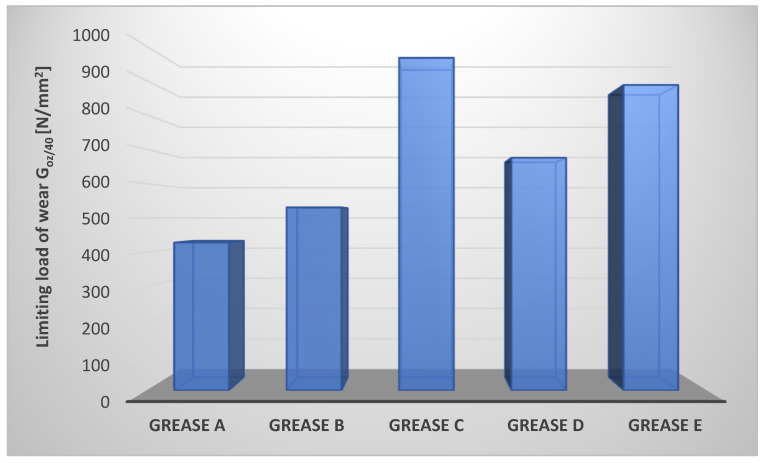
Limiting load of wear of tribosystem lubricated with compositions produced on rapeseed oil with different thickeners.

The anti-wear properties of the tested vegetable lubricants were verified by determining the limiting load of wear G_oz/40_ of the tribosystem lubricated with the evaluated compositions. The results are shown in Figure 5.

Tests of the lubricating properties of the produced lubricating compositions showed that the used thickeners change the ability of the lubricants to protect the tribosystem against wear. The durability of the boundary layer was indicated by the wear index G_oz/40_. The higher the index, the greater the durability of the boundary layer and the reduction of wear. The lubricating composition with calcium stearate as a dispersed phase has the highest wear index. The composition E, in which the layered silicate–montmorillonite was in the dispersed phase, was characterized by a comparable value of the G_oz/40_ parameter to the composition C. On the other hand, the lowest value of this parameter was observed for lubricant A, in which the lithium stearate was used as a dispersed phase. The value of the limiting load of wear for composition B was 23.9 [%] higher and for composition D was 57.4 [%] higher than the value of this parameter for composition A in which the lithium stearate was used as a dispersed phase. In addition, the montmorillonite-thickened grease composition has 106.6 [%] higher anti-wear protection than the vegetable grease-thickened lithium stearate. The composition in which the calcium stearate (grease C) was used as a dispersed phase showed very good anti-wear properties, 125 [%] higher than the composition thickened with lithium soap.

It was found that the use of both soap and inorganic thickeners as a dispersed phase of vegetable lubricating greases has a favorable effect on the anti-wear properties of the tested lubricating compositions. The greatest anti-wear durability was obtained by using calcium stearate as a dispersed phase of the produced lubricating compositions, which makes it possible to obtain a grease characterized by the highest value of the G_oz/40_ parameter. Thus, the type of dispersed phase used in the composition of vegetable lubricants has an effect on the value of the limiting load of wear of the tested lubricating compositions. A comparison of the anti-wear properties of lubricating compositions in which soap thickeners and amorphous silica and montmorillonite were used as a dispersed phase, prepared on the base of vegetable oils, with compositions prepared on the base of synthetic or mineral oils with the same dispersed phase, indicates that the use of vegetable oils as the base oil for lubricants determines effective anti-wear protection, which cannot be said for lubricants prepared on the base of synthetic or mineral oils.

Lubricant quality criteria, particularly for the food industry, are set individually by machine and device manufacturers. As a result of the market analysis, it can be stated that the lubricating compositions with G_oz/40_ > 600 [N/mm^2^] have very good anti-wear properties, those with the limiting load of wear in the range of 400–600 [N/mm^2^] provide effective anti-wear protection, whereas, if G_oz/40_ < 400 [N/mm^2^], then we speak of insufficient anti-wear properties. The level of anti-wear properties of all obtained lubricating compositions makes them effective lubricants under constant load conditions.

### 3.1. Tests of Rheological Properties of Lubricating Greases Carried Out with Classical Rheometer

**Figure 6:**
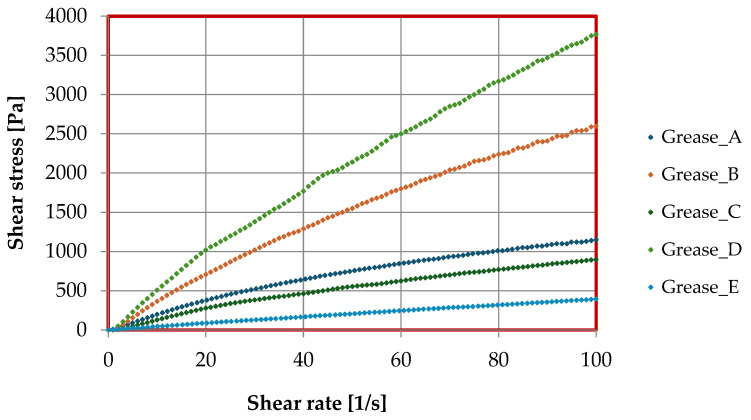
The dependence of shear stress on shear rate (flow curves) for lubricants produced on different thickeners.

**Figure 7:**
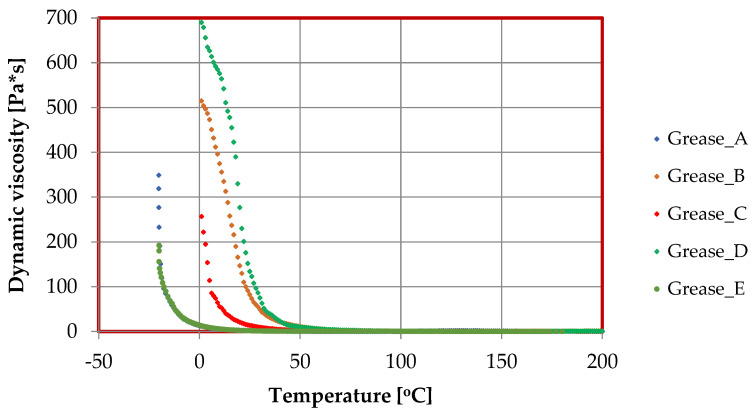
The dependence of dynamic viscosity from temperature (viscosity curves) for greases produced with different thickeners.

**Figure 8:**
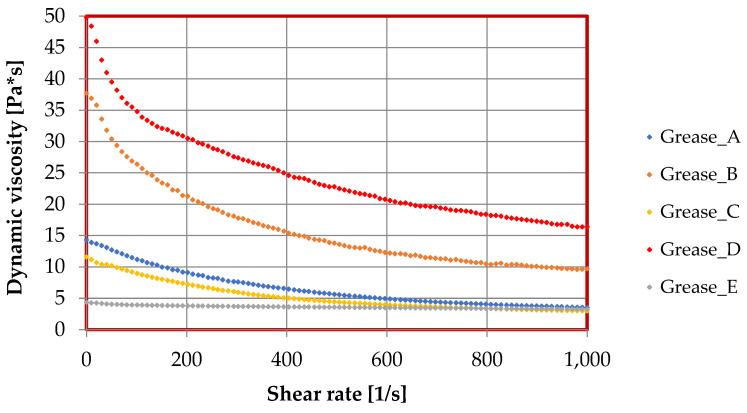
The dependence of dynamic viscosity from shear rate (viscosity curves) for lubricants produced with different thickeners.

Flow curves and viscosity curves were determined for the prepared lubricant compositions using an MCR 101 rotational rheometer. The effects of the thickeners used in the tested lubricants on the rheological properties are shown in Figure 6, Figure 7 and Figure 8, while the parameters of the Casson model describing the lubricant flow curves are given in Table 2.

On the basis of the data obtained from the flow curves of the tested lubricant formulations and the rheological parameters determined using the Casson rheological model, it can be concluded that the use of different thickeners to formulate the lubricant formulations significantly altered the rheological properties of the tested vegetable lubricants. In all cases, a very good fit of the data obtained to the Casson model was observed at 97 ÷ 99 [%]. The lowest structural viscosity was found for grease E thickened with montmorillonite, with similar viscosity values for compositions A and C, in which soap thickeners (lithium and calcium) were the dispersed phase. Much higher values of this parameter were observed for composition B, where aluminum stearate was the dispersed phase, while the highest value of structural viscosity was observed for composition D, where Aerosil amorphous silica was the thickener. The composition thickened with amorphous silica had the highest yield point value, while the composition in which montmorillonite was the dispersed phase had the lowest value. Lubricants thickened with lithium stearate and calcium stearate were characterized by similar yield point values, while the composition in which the calcium stearate was used as the thickener had a yield point value of 21.9 [Pa], which was intermediate between the highest and lowest values for the tested lubricating compositions.

The higher value of both yield point and structural viscosity for composition B thickened with aluminum stearate in compared to the other compositions produced on soap thickeners is due to the fact that the chemical structure of the aluminum soap allows modification of the chemical structure of the creating grease and the interaction of the aluminum thickener with the main chain of the rapeseed oil base is effective because decrease the distances between the individual molecules of the oil base, causing a synergistic interaction between the oil base and the aluminum stearate. Major differences were also observed between the discussed parameters for the compositions thickened of montmorillonite and Aerosil amorphous silica. The significant differences between the rheological properties of these two lubricating compositions are due to the different chemical structures of the two used thickeners. The amorphous silica was modified with dimethylchlorosilane, causing a more efficient attachment to the rapeseed oil molecules; moreover, the distance between the molecules was reduced, and van der Waals forces were stronger than with montmorillonite. Thanks to this phenomenon, there is a stronger binding of the thickener molecules to the chain of the vegetable oil base and a change in the chemical structure of the creating grease, which causes an improvement of the functional properties of the created grease, including rheological properties.

A change in shear stress with increasing shear rate was observed for the tested lubricating compositions, depending on the type of used thickener. For grease A, thickened with lithium stearate, the shear stress reached a value of 1140 [Pa]; for composition B, in which aluminum stearate was the dispersed phase, the stress value was 2600 [Pa]. For composition C, the stress value was 892 [Pa], while for composition D, the stress value was 3770 [Pa]. For composition E, which was thickened with montmorillonite, it was 390 [Pa]. The difference in the stress value between the different compositions is due to the different structures of the used thickeners and their more effective incorporation into the chain of the vegetable oil base, which leads to a change in the chemical structure of the obtained lubricants and, consequently, to significant changes in the individual rheological parameters.

A study of the effect of viscosity on temperature for lubricating compositions prepared with different thickeners is shown in Figure 7. Dynamic viscosity decreases with temperature, which is characteristic of lubricants. No significant differences in dynamic viscosity were found for the lubricating compositions tested at temperatures above 50 [°C]. The greatest changes in viscosity were observed at sub-zero temperatures, which is particularly important when working on lubricants in those industries where freezing temperatures are encountered. In these cases, it is important that the viscosity at freezing temperatures has the lowest value, as the effective work of the lubricant depends on the viscosity. The lower the viscosity, the more effective the lubricant’s work to effectively protect machinery and equipment from immobilization.

The most favorable rheological properties were observed for composition C, which was produced using calcium stearate, as it allows the lowest dynamic viscosity at −20 [°C]. Lubricants produced with lithium stearate or aluminum stearate were characterized by higher viscosity at low temperatures. The highest viscosity at freezing temperatures was observed for a composition thickened with amorphous silica, while a much lower viscosity was observed for a lubricating composition in which montmorillonite was used as a thickener. The change in the rheological behavior of the individual lubricating compositions was due to the different structures of the individual thickeners. The way in which the thickener is incorporated into the lubricant structure formed by the oil base particles and the thickener particles change the functional properties of the created lubricant. The change of lubricant properties is particularly characteristic of the different types of the dispersed phase, which is largely responsible for the tribological and rheological behavior of the lubricant. Some thickeners are particularly resistant to changes in rheological properties especially at low temperatures. This is due to their chemical structure, namely, the presence in their chemical composition of reactive atoms (e.g., aluminum or silicon) which, by attaching themselves to the vegetable oil chain, increase the resistance of the produced lubricant to mechanical-temperature forcing.

Tests were also carried out on the dependence of dynamic viscosity on the shear rate for the lubricating compositions. The results are shown in Figure 8. As the shear rate increased, the dynamic viscosity of the tested lubricating compositions decreased. The change in lubricant viscosity was due to the use of different thickeners in the formulation of the lubricating composition.

The type of dispersed phase used had a significant effect on the change of dynamic viscosity of the vegetable greases. The highest viscosity value was recorded for composition D, which was produced with amorphous silica. The lower viscosity value was found for grease B, where the aluminum stearate was used as a thickener. Lubricants based on rapeseed oil and thickened with calcium and lithium stearate have a similar dynamic viscosity value, while the lowest viscosity value was observed for composition E, where montmorillonite was used as a dispersed phase. The differences in the viscosity results between the different lubricants are due to the use of different types of dispersed phases. The used thickeners varied considerably, particularly in terms of chemical structure. The way in which the thickener molecules are incorporated into the base oil chain causes a significant change in the rheological parameters of the created lubricating compositions. Some thickeners are extremely rheologically effective, particularly amorphous silica, montmorillonite, and aluminum stearate. The chemical structure of these three thickeners means that. when they are introduced into the vegetable oil base, the distance between the oil and thickener molecules is reduced, the chemical structure of the created grease is stabilized, and the resistance of the structure of the created greases to deformation is increased, thereby significantly improving to the rheological properties of vegetable lubricants.

### 3.2. Tests of Rheological Properties of Lubricating Greases Carried Out with Optical Rheometer

The dependence of the MSD correlation function on time and the dependence of the G′ and G″ modulus values on frequency were carried out for the produced lubricating compositions. The test results of the mentioned dependencies are shown in Figure 9, Figure 10, Figure 11, Figure 12, Figure 13, Figure 14, Figure 15, Figure 16, Figure 17 and Figure 18.

The rheological properties of vegetable grease, in which the lithium stearate was the dispersed phase were determined using an optical rheometer. The carried out dependence of MSD correlation function on time shows that the test lubricant was characterized by the occurrence of two growth phases. The first, from 0 to 1 [s], and the second, from 1 to 10 [s], in which a sudden increase in the value of the MSD function occurs, characterizing the viscosity of the tested lubricant sample. Based on the available data, the value of the elasticity index EI was determined, which characterizes the strength of the viscoelastic properties of tested grease. The higher the value of the elasticity index, the weaker the viscoelastic properties. For grease A, in which the lithium stearate was used as a thickener, the value of elasticity index EI was 9.92 × 10^6^ [µm^−2^], which indicates the weak viscoelastic properties of tested grease and a greater tendency to change structure under the influence of applied forces.

**Figure 10:**
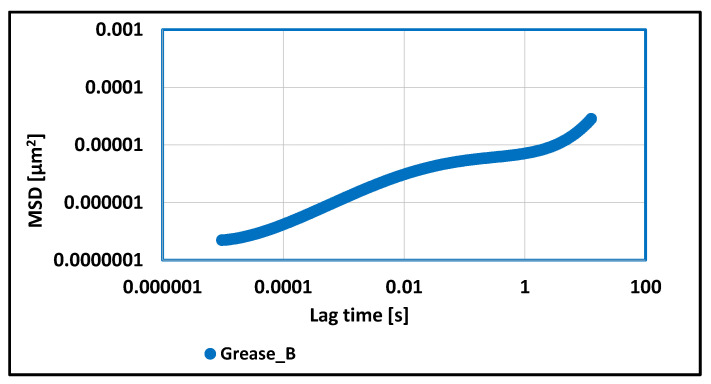
The dependence of the value of the MSD correlation function on time for a lubricating composition based on rapeseed oil and thickened with aluminum stearate.

In Figure 10, the three phases of the MSD correlation function can be observed. The first phase shows an increase of up to 0.05 [s], followed by a plateau phase, where the value of the MSD function does not change, and a third phase, where the value of the MSD function increases again. More than 15 times lower values of the MSD function were observed, and the calculated elasticity index value was 5.53 × 10^6^ [µm^−2^], which proves the stronger viscoelastic properties of the aluminum stearate grease in relation to grease thickened with the lithium stearate. A consequence of such test results is the greater resistance of this grease to structural changes under the influence of applied mechanical forces.

A change in viscoelastic properties was observed in vegetable grease composition, where the calcium stearate was used as a thickener in relation to greases thickened with lithium or aluminum stearate. In this case, the three phases of the MSD function were also observed, i.e., the first growth phase, the plateau phase, and the second growth phase. The value of the MSD correlation function reached values approximately 100 times lower than the value of this function obtained for grease thickened with lithium stearate. Such results indicate about the much stronger viscoelastic properties of the tested grease sample. The determined value of the elasticity index was 1.2 × 10^6^ [µm^−2^], which indicates much stronger viscoelastic properties in relation to the two previous greases in which the soap thickeners were used. The stronger viscoelastic properties of the tested grease caused an increase in the stability of the evaluated grease against mechanical stress and are characterized by a lower tendency to change structure during work under extreme conditions.

A vegetable grease, in which the amorphous silica–Aerosil 300 was used as a thickener, showed different viscoelastic properties to those previously discussed. For carrying out the correlation function MSD on time, the lower values of this parameter were observed in relation to greases thickened with lithium or aluminum stearate, and the higher values in relation to grease thickened with calcium stearate. In the case of evaluated grease, the three phases of the MSD correlation function were observed, namely, a growth phase, a plateau phase, and a second growth phase. In this case, the elasticity index value was also determined. This value was 5.23 × 10^6^ [µm^−2^] and was lower than for greases thickened with lithium and aluminum stearate, indicating its stronger viscoelastic properties in relation to these two greases. Grease thickened with calcium stearate has better viscoelastic properties (lower elasticity index value) than the grease produced with different soap thickeners. From the obtained results, it can be concluded that the evaluated grease is resistant to structural changes under the influence of changing working conditions in steel friction joints.

Grease composition prepared on the vegetable oil base and thickened with montmorillonite was characterized by different viscoelastic properties in relation to the lubricating compositions previously discussed, which indicates that the type of thickener had a decisive importance for the level of rheological properties of tested vegetable lubricating compositions. In the course of the MSD correlation function, the occurrence of three phases was observed, i.e., a growth phase, a plateau phase, and a second growth phase. The curve is very flattened, which may be related to the structure of the used thickener, i.e., montmorillonite. The values of MSD function are much lower than for the grease thickened with lithium stearate and comparable to those obtained for the grease thickened with aluminum stearate and Aerosil. The value of elasticity index EI for this grease composition was 2.64 × 10^6^ [µm^−2^] and was lower than most of the tested grease compositions without the composition, in which the calcium stearate was used as a thickener. Such results testify to moderately strong viscoelastic properties, which leads to the conclusion that the produced lubricant was a stable substance on the changes of the chemical structure under the influence of variable conditions prevailing during work in tribological friction joints.

The dependence of the elastic modulus G′ and the viscous modulus G″ on frequency were determined for studied lubricating compositions.

For grease produced on vegetable oil and thickened with lithium soap, it was noted that, at low-frequency values up to 1 [rad/s], the G″ modulus dominates, characterizing the viscous properties of the tested medium. In the next frequency range up to 5000 [rad/s], the elastic modulus dominates, then, up to 90,000 [rad/s], the viscous modulus takes higher values, so that, above the limit of 90,000 [rad/s], the elastic modulus G′ again obtains higher values. Thus, depending on the magnitude of the extortions to which the lubricant sample is subjected, the lubricant under test behaves either as a liquid (higher values of the elastic modulus) or as a solid (higher values of the viscous modulus).

**Figure 15:**
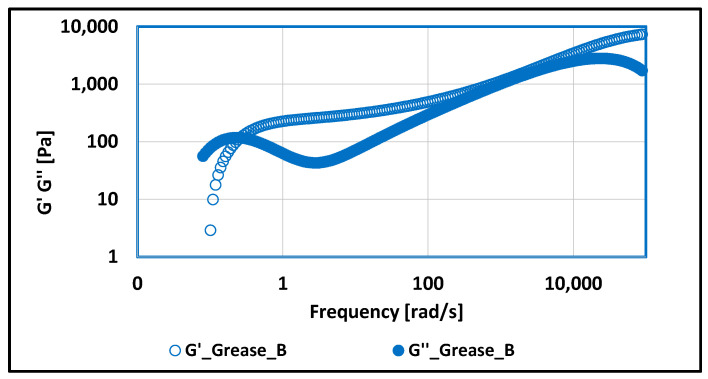
The dependence of G′ and G″ modulus values on frequency for grease produced on rapeseed oil base and thickened with aluminum stearate.

The use of aluminum soap as a thickener for vegetable lubricating grease caused significant changes in the rheological properties compared with the lubricating composition produced from vegetable oil and thickened with lithium soap. For grease B (Figure 15), it was observed that, in the very low-frequency range (up to 0.22 [rad/s]), the viscous characteristics predominate. Then, as the frequency increases up to a value of 100,000 [rad/s], the higher values are adopted of the G′ modulus characterizing the elastic–fluid–characteristics. In this way, the tested lubricating composition is diluted under the influence of the applied mechanical forces. The characteristics of the tested lubricant were changed towards the liquid state, causing a change in the chemical structure; however, there was no complete degradation of the tested lubricant because, across the range of frequency, the lubricating composition was characterized by viscoelastic properties. Thus, depending on the extortions, to which the lubricant was subjected, its properties tend to be either a solid or a liquid.

For vegetable grease thickened with calcium soap, changes in rheological properties were observed in relation to greases thickened with lithium stearate or aluminum stearate. For the discussed grease composition, the G″ modulus dominates up to a frequency of 0.68 [rad/s], characterizing the viscous properties of the tested grease (high structural stability, high resistance to change). Above the 0.68 [rad/s], the elastic properties, characteristic of liquids, dominate. This behavior of the tested composition indicates that under the influence of mechanical extortions, there is a dilution process and a decrease in viscosity, which can lead to a slow degradation of the structure and changes in the properties tested lubricant.

For rapeseed grease thickened with amorphous silica, frequency-dependent changes in rheological properties were observed. In the frequency range of 0.01 ÷ 0.39 [rad/s], the G″ modulus, which characterizes the viscous properties, similar to those of a solid, adopts higher values. The application of mechanical forces causes a change in the structure of the tested lubricating composition, as elastic properties predominate up to a frequency of 100,000 [rad/s]. The mechanical forces to which the lubricant is subjected during work in the friction node cause changes in the structure, the viscosity decreases, the substance is diluted under the effect of shear forces, and it tends toward the structure represented by liquids, but does not completely lose its characteristics; therefore, it is still a plastic lubricant; however, its consistency changes.

Significant changes in rheological properties were observed in lubricating compositions thickened with montmorillonite, a representative of layered silicates, compared to compositions, where soap thickeners were used. The rheological properties are influenced by the chemical structure of the used thickener. Up to a frequency value of 0.30 [rad/s], the viscous properties, represented by higher values of G′ modulus, dominate. Above a frequency value of 0.30 [rad/s] and up to 50,000 [rad/s], the elastic characteristics predominate because the elastic modulus value G′ is higher than the viscous modulus G″. The applied load to which it was subjected to grease during the test caused a reduction in viscosity, i.e., a dilution of the structure, while maintaining the other properties of the grease, both tribological and physico-chemical. The applied load caused a change in the consistency class of tested lubricating composition but did not lead to complete degradation of the grease. Higher values of the modulus of elasticity—tending towards the properties of a liquid—as a result of mechanical forcing (shear forces) cause a change in the lubricant consistency and an increase in the tendency to change the chemical structure of the tested lubricating composition but did not cause significant changes of the essential properties of the grease.

## 4. Conclusions

Compositions, in which the amorphous silica–Aerosil and montmorillonite were used as a dispersed phase were characterized by very good anti-scuffing properties under conditions of abruptly increasing tribosystem load. Other compositions were characterized by lower values of this parameter. The highest level of anti-scuffing properties under scuffing conditions was observed in greases prepared on the base of rapeseed oil and thickened with amorphous silica–Aerosil and montmorillonite, while compositions in which a stearate (lithium, aluminum, and calcium) were a dispersed phase did not provide satisfactory anti-scuffing protection. Under conditions of linearly increasing tribosystem load, the most effective anti-scuffing protection represented those lubricating compositions in which amorphous silica and montmorillonite were used as a dispersed phase. Other compositions, in which soap thickeners were used as a dispersed phase were characterized by a similar level of this parameter but lower than compositions, in which were used thickeners containing silicon in their composition. The application of calcium stearate and montmorillonite as a dispersed phase of the tested lubricating greases causes an increase in the value of the G_oz_ parameter compared to compositions thickened with lithium stearate, aluminum stearate, and amorphous silica, which indicates about high resistance of the tested lubricating compositions thickened with calcium soap and montmorillonite on interruption of boundary layer.

The higher value of both yield point and structural viscosity for composition thickened with aluminum stearate compared to the other compositions produced on soap thickeners is due to the fact that the interaction of the aluminum thickener with the main chain of the rapeseed oil base is effective because decrease the distances between the individual molecules of the oil base, causing a synergistic interaction between the oil base and the aluminum stearate. Major differences were also observed between the discussed parameters for the compositions thickened of montmorillonite and Aerosil amorphous silica. The significant differences between the rheological properties of these two lubricating compositions are due to the different chemical structures of the two thickeners used. The amorphous silica was modified with dimethylchlorosilane, causing a more efficient attachment to the rapeseed oil molecules, in which the distance between the molecules was reduced and the van der Waals forces were stronger than with montmorillonite. Thanks to this phenomenon, there is a stronger binding of the thickener molecules to the chain of the vegetable oil base and a change in the chemical structure of the creating grease, which causes an improvement in the functional properties of the created grease, including rheological properties.

A change in shear stress with increasing shear rate was observed for the tested lubricating compositions, depending on the type of used thickener. The difference in the stress value between the different compositions is due to the different structures of the used thickeners and their more effective incorporation into the chain of the vegetable oil base, which leads to a change in the chemical structure of the obtained lubricants and, consequently, to significant changes in the individual rheological parameters.

The change in the rheological behavior of the individual lubricating compositions was due to the different structures of the individual thickeners. The way in which the thickener is incorporated into the lubricant structure formed by the oil base particles and the thickener particles change the functional properties of the created lubricant. The change of lubricant properties is particularly characteristic of the different types of the dispersed phase, which is largely responsible for the tribological and rheological behavior of the lubricant. Some thickeners are particularly resistant to changes in rheological properties especially at low temperatures. This is due to their chemical structure, namely the presence in their chemical composition of reactive atoms (e.g., aluminum or silicon) which, by attaching themselves to the vegetable oil chain, increase the resistance of the produced lubricant to mechanical-temperature forcing.

For grease, in which the lithium stearate was used as a thickener, the value of the elasticity index determines the weak viscoelastic properties of tested grease and a greater tendency to change structure under the influence of applied forces. For vegetable grease thickened with aluminum stearate, more than 15 times lower values of the MSD function were observed, and the calculated elasticity index value proves the stronger viscoelastic properties of the aluminum stearate grease in relation to grease thickened with the lithium stearate. A consequence of such test results is the greater resistance of this grease to structural changes under the influence of applied mechanical forces. The determined value of the elasticity index for grease thickened with calcium stearate indicates about much stronger viscoelastic properties in relation to the two previous greases in which the soap thickeners were used. The stronger viscoelastic properties of the tested grease caused an increase in the stability of the evaluated grease against mechanical stress and are characterized by a lower tendency to change structure during work under extreme conditions. The elasticity index value for grease thickened with amorphous silica was lower than for greases thickened with lithium and aluminum stearate, indicating its stronger viscoelastic properties in relation to these two greases.

## Figures and Tables

**Figure 9:**
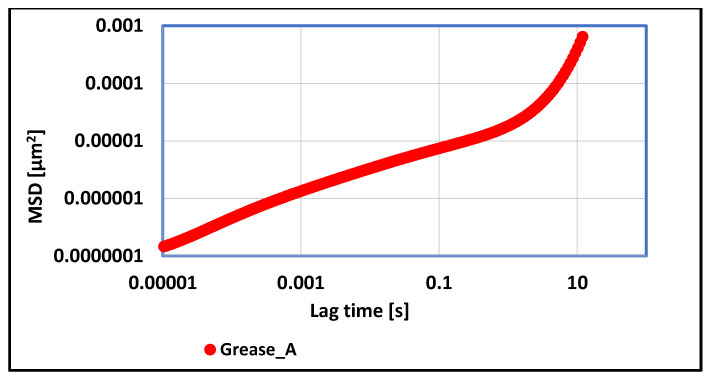
The dependence of the value of the MSD correlation function on time for a lubricating composition based on rapeseed oil and thickened with lithium stearate.

**Figure 11:**
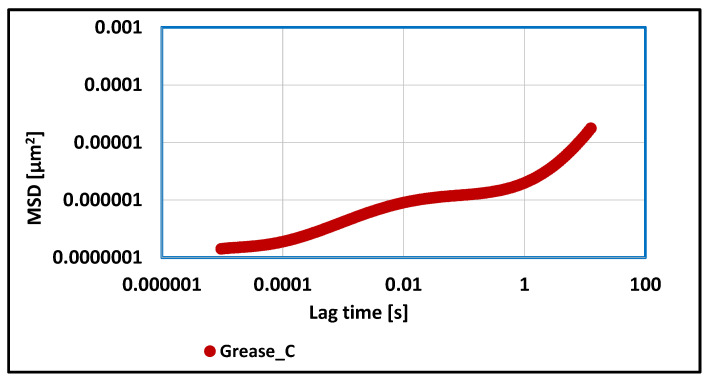
The dependence of the value of the MSD correlation function on time for a lubricating composition based on rapeseed oil and thickened with calcium stearate.

**Figure 12:**
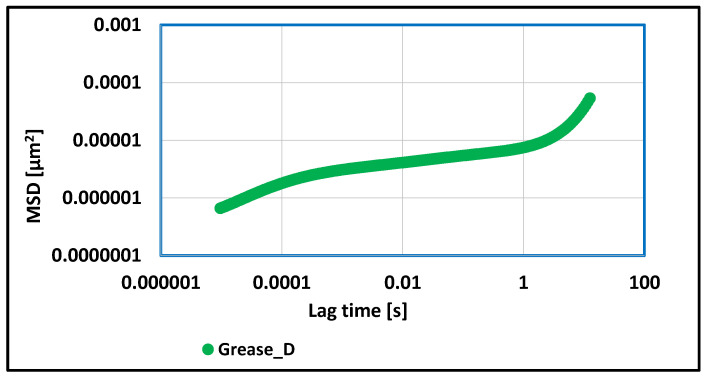
The dependence of the value of the MSD correlation function on time for a lubricating composition based on rapeseed oil and thickened with amorphous silica–Aerosil.

**Figure 13:**
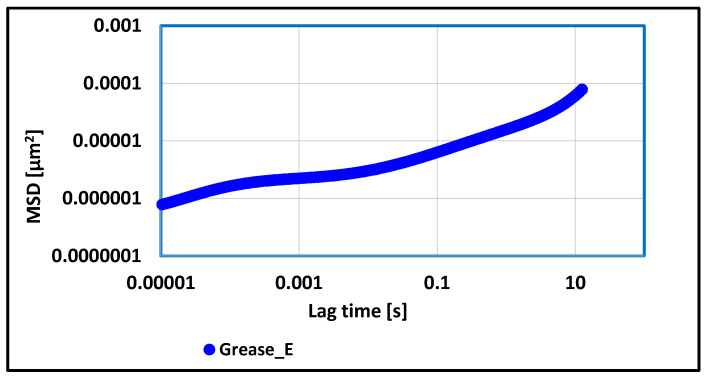
The dependence of the value of the MSD correlation function on time for a lubricating composition based on rapeseed oil and thickened with montmorillonite.

**Figure 14:**
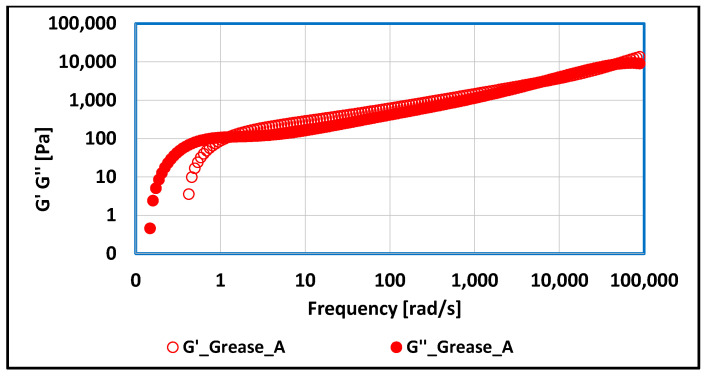
The dependence of G′ and G″ modulus values on frequency for grease produced on rapeseed oil base and thickened with lithium stearate.

**Figure 16:**
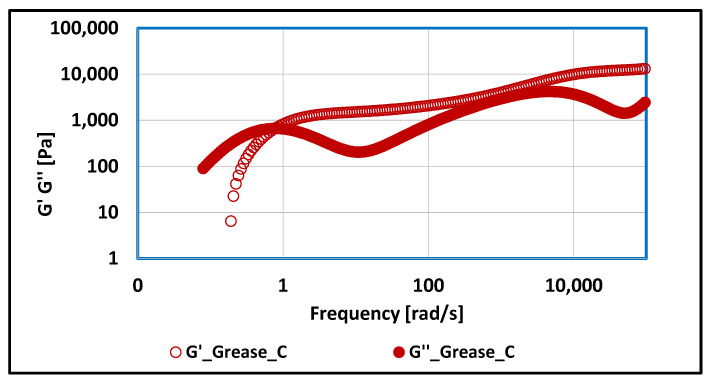
The dependence of G′ and G″ modulus values on frequency for grease produced on rapeseed oil base and thickened with calcium stearate.

**Figure 17:**
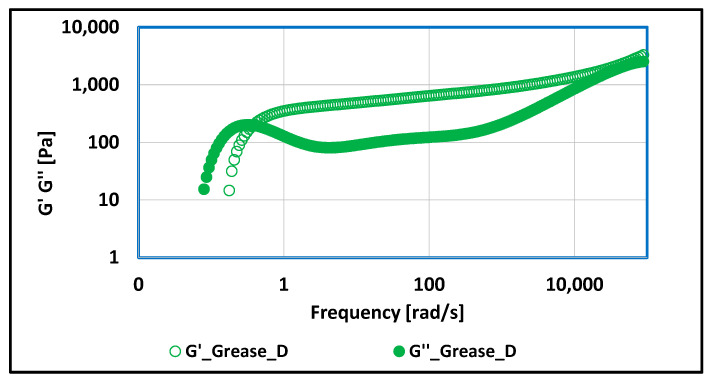
The dependence of G′ and G″ modulus values on frequency for grease produced on rapeseed oil base and thickened with amorphous silica–Aerosil.

**Figure 18:**
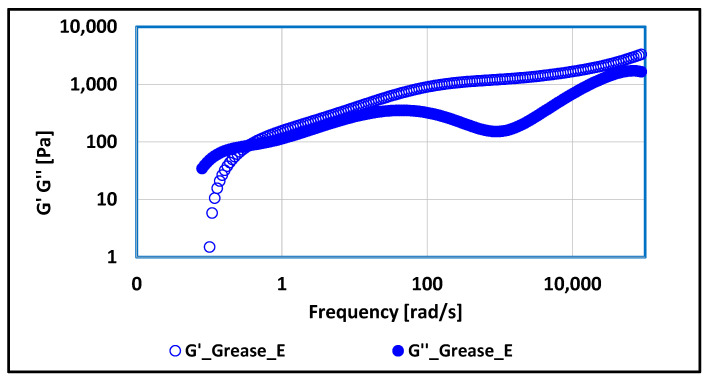
The dependence of G′ and G″ modulus values on frequency for grease produced on rapeseed oil base and thickened with montmorillonite.

**Table 1:** Chemical composition of tested vegetable lubricants.

Designation of the Test Grease	Dispersion Phase	Dispersed Phase	Penetration [mm/10]
A	Rapeseed oil (92%)	Lithium stearate (8%)	279.00
B	Rapeseed oil (93%)	Aluminum stearate (7%)	267.75
C	Rapeseed oil (91%)	Calcium stearate (9%)	271.50
D	Rapeseed oil (90%)	Silica Aerosil (10%)	294.00
E	Rapeseed oil (88%)	Montmorillonite (12%)	286.50

**Table 2:** Casson’s rheological model parameters for the tested lubricating compositions.

Identification of Test Grease	Casson Model		
	Yield Point *τ*_0_ [Pa]	Correlation Coefficient R^2^ [-]	Structural Viscosity *η*_∞_ [Pa*s]
Grease A	9.0	0.981	9.3
Grease B	21.9	0.974	24.6
Grease C	6.1	0.985	6.2
Grease D	48.5	0.968	65.7
Grease E	4.6	0.991	4.5

## Data Availability

Data are contained within the article.
